# Compressed sensing based dynamic MR image reconstruction by using 3D-total generalized variation and tensor decomposition: k-t TGV-TD

**DOI:** 10.1186/s12880-022-00826-1

**Published:** 2022-05-27

**Authors:** Jucheng Zhang, Lulu Han, Jianzhong Sun, Zhikang Wang, Wenlong Xu, Yonghua Chu, Ling Xia, Mingfeng Jiang

**Affiliations:** 1 Department of Clinical Engineering, The Second Affiliated Hospital, School of Medicine, Zhejiang University, Hangzhou, 310019 People’s Republic of China; 2 School of Information Science and Technology, Zhejiang Sci-Tech University, Hangzhou, 310018 People’s Republic of China; 3 Zhejiang Aerospace HengJia Data Technology Co., Ltd., Jiaxing, People’s Republic of China; 4 Department of Radiology, The Second Affiliated Hospital, School of Medicine, Zhejiang University, Hangzhou, 310027 People’s Republic of China; 5 Department of Biomedical Engineering, China Jiliang University, Hangzhou, 310018 People’s Republic of China; 6 Department of Biomedical Engineering, Zhejiang University, Hangzhou, 310027 People’s Republic of China

**Keywords:** Dynamic cardiac MR imaging, Higher-order singular value decomposition, Total generalized variation, Sparse representation

## Abstract

**Purpose:**

Compressed Sensing Magnetic Resonance Imaging (CS-MRI) is a promising technique to accelerate dynamic cardiac MR imaging (DCMRI). For DCMRI, the CS-MRI usually exploits image signal sparsity and low-rank property to reconstruct dynamic images from the undersampled k-space data. In this paper, a novel CS algorithm is investigated to improve dynamic cardiac MR image reconstruction quality under the condition of minimizing the k-space recording.

**Methods:**

The sparse representation of 3D cardiac magnetic resonance data is implemented by synergistically integrating 3D total generalized variation (3D-TGV) algorithm and high order singular value decomposition (HOSVD) based Tensor Decomposition, termed *k-t* TGV-TD method. In the proposed method, the low rank structure of the 3D dynamic cardiac MR data is performed with the HOSVD method, and the localized image sparsity is achieved by the 3D-TGV method. Moreover, the Fast Composite Splitting Algorithm (FCSA) method, combining the variable splitting with operator splitting techniques, is employed to solve the low-rank and sparse problem. Two different cardiac MR datasets (cardiac perfusion and cine MR datasets) are used to evaluate the performance of the proposed method.

**Results:**

Compared with the state-of-art methods, such as *k-t* SLR, 3D-TGV, HOSVD based tensor decomposition and low-rank plus sparse method, the proposed *k-t* TGV-TD method can offer improved reconstruction accuracy in terms of higher peak SNR (PSNR) and structural similarity index (SSIM). The proposed *k-t* TGV-TD method can achieve significantly better and stable reconstruction results than state-of-the-art methods in terms of both PSNR and SSIM, especially for cardiac perfusion MR dataset.

**Conclusions:**

This work proved that the *k-t* TGV-TD method was an effective sparse representation way for DCMRI, which was capable of significantly improving the reconstruction accuracy with different acceleration factors.

## Introduction

In Magnetic Resonance Imaging (MRI), imaging speed is limited by slow acquisition of full k-space using magnetic field gradients [1]. Minimizing the k-space recording time without compromising image quality has been a main thrust of MR imaging research. With the advent of compressed sensing (CS) theory [2, 3], MR image reconstruction with sparsity-promoted regularization (e.g., *ℓ*_1_-based regularization), termed as CS-MRI [4–10], has gained popularity for its high imaging speed. The effective exploitation of the signal sparsity enables the MR image reconstruction from far fewer k-space samples possible than conventional methods require, thus CS-MRI can significantly reduce the scan time. The compressed sensing theory has been successfully applied to both static and dynamic magnetic resonance imaging (dMRI) reconstructions [11–14].

In CS-MRI, the method used to sparsify the MR image plays an important role in the image reconstruction. The most used sparsity bases are predefined mathematical transforms, such as discrete cosine transform (DCT), and discrete wavelet transform (DWT). Recently, the singular value decomposition (SVD) method has been used as a data-adaptive sparsity basis in CS-MRI reconstruction [15, 16], and it has been found that the SVD-based method could significantly accelerate the reconstruction process and achieve better image quality than those commonly used sparsifying transforms (DCT and DWT). Majumdar et al. proposed to exploit the nuclear norm regularization to implement the CS-MRI reconstruction, and the results showed that the proposed reconstruction method was faster than other methods [6]. In addition, the linear combination of Total Variation (TV) and wavelet sparse regularization, known as TV-L1 problem, is very popular in many CS-MRI models [5, 6, 17], which can be considered as processing the MR image to be sparse by both the specific transform and finite-differences at the same time. Due to the stair-case artifacts caused by the conventional TV-based regularization [18, 19], several generalizations and extensions of TV have been introduced to improve the CS-MRI reconstruction accuracy, such as Total Generalized Variation (TGV) [18–20], Higher Degree Total Variation (HDTV) [21]. Nonlocal Total Variation (NLTV) [22–24] is another effective way to address the issue of stair-case artifacts. Although effective in practice, it involves higher computational complexity than the conventional TV method.

For dynamic MR image reconstruction, Ji, et al*.* adopted the difference between the reconstructed image and the reference image to represent the spatial sparsity [25]. However, when compared with the reference frame, the sparsity of the difference image got worse with the increase of the subsequent frame distance. To solve this problem, Majumdar took the difference between two adjacent sub-images as a sparse representation of the reconstructed MR image [13]. In addition, Usman put forward the concept of a sparse group of dynamic MRI, utilizing both MRI signal itself sparsity and the group structure information between signals [26, 27], which can effectively improve the image reconstruction quality. Moreover, a novel blind compressed sensing frame work was proposed to recover dynamic magnetic resonance images from undersampled measurements [28, 29], which has been proved to provide superior reconstruction performance in comparison to existing low rank and compressed sensing schemes. Recently, *k-t* SLR (*k-t* Sparisity and Low-Rank) method has been proposed to accelerate dynamic MRI by exploiting sparsity and low rank properties of the image data [30, 31]. To exploit the low-rank structure, the *k-t* SLR method reshaped the 3D dataset into a large 2D matrix through a two-step process: vectorize the 2D images in a dynamic sequence first and then concatenate them to form a matrix. In most of the existing dynamic CS-MRI methods, 2D/1D transforms were applied to solve the 3D dynamic problem, which, by treating the 3D data as a series of 2D images, unfolded the 3D dataset into a 2D matrix to explore the spatiotemporal redundancy [30–33]. In addition, Majumdar [34, 35] acted the dynamic MR image reconstruction problem as a least squares minimization regularized by *lp*-norm as the sparsity penalty and Schatten-*q* norm as the low-rank penalty sparsity, which can yield much better reconstruction results than *k-t* SLR method. However, reshaping a high-order tensor into a matrix or vector may neglect the inherent data redundancy, thus greatly degrading the reconstructed image quality. To promote the signal sparsity representation by exploring the redundancy of the high-dimension data format, Yu et al. proposed tensor decomposition-based sparsifying transform, that is, high-order Singular Value Decomposition (HOSVD) [36], which can outperform the conventional sparse recovery methods for high-dimensional cardiac imaging reconstruction accuracy given the same amount of k-space data set [37].

In this paper, we will further improve the HOSVD based CS-MRI method to synergistically integrate 3D-TGV algorithm and HOSVD-based Tensor Decomposition, termed as *k-t* TGV-TD method. In the proposed method, the low rank structure of the 3D dynamic cardiac MR data is performed by the HOSVD method, and the localized image sparsity is achieved by the 3D-TGV method. Meanwhile, the Fast Composite Splitting Algorithm (FCSA) method [6], combining the variable splitting with operator splitting techniques, is employed to solve the low-rank and sparse problem [38]. Two different cardiac MR datasets (cardiac perfusion and cine MR datasets) are used to evaluate the performance of the proposed method.

## Theory of k-t TGV-TD method

In the proposed CS-MRI technique, 3D-TGV and HOSVD based tensor decomposition are used to promote the sparsity of the dynamic MR signals, and the *k-t* TGV-TD optimization problem can be formed as:

1 $$\mathop {\arg \min }\limits_{\chi } \left\{ {\left\| {A_{u} (\chi ) - b} \right\|_{2}^{2} + \lambda_{1} \cdot TGV_{\alpha }^{2} (\chi ) + \lambda_{2} \varphi (\chi )} \right\}$$

where $A_{u}$ is undersampled Fourier operator of the MR image, ***b*** is undersampled measurement of *k*-space data, and $\chi$ is a third order tensor used to represent the spatial–temporal 3D cardiac MR data. $\lambda_{1} \;{\text{and}}\;\lambda_{2}$ are two positive regularization parameters that determine the trade-off between the data consistency and the sparsity regularization terms. $\varphi (\chi )$ is the tensor decomposition, and $TGV_{\alpha }^{2} (\chi )$ is the second order TGV penalty function.

Fast composite splitting algorithm processes the original and composite regularization problem into two simpler sub-problems, which are then solved by using the fast iterative shrinkage-threshold algorithm (FISTA). In this way, we could finally reconstruct the dynamic images via an iterative combination [39]. Specifically, the complex composite reconstruction problem in Eq. (1) can be decomposed into two simpler regularization subproblems, that is, TGV subproblem and TD subproblem, as shown in Eqs. (2) and (3):

2 $$\mathop {\arg \min }\limits_{\chi } \left\{ {\frac{1}{2}\left\| {A_{u} (\chi ) - b} \right\|_{2}^{2} + \lambda_{1} \cdot TGV_{\alpha }^{2} (\chi )} \right\}$$

3 $$\mathop {\arg \min }\limits_{\chi } \left\{ {\frac{1}{2}\left\| {A_{u} (\chi ) - b} \right\|_{2}^{2} + \lambda_{2} \varphi (\chi )} \right\}$$

The basic idea of FISTA is to build regularization for the linearized differentiable part of the objective function in each iteration [38–40]. Therfore, the subproblem Eqs. (2) and (3) can be extended into two parts respectively:

4 $$\min \left\{ {F(\chi ) \equiv f(\chi ) + g(\chi ):\chi \in C^{{I_{1} \times I_{2} \times I_{3} }} } \right\}$$

where $f(\chi ) = \frac{1}{2}\left\| {A{}_{u}(\chi ) - b} \right\|_{2}^{2}$ is a smooth convex function which is continuously differentiable with Lipschitz constant ***L***_***f***_ (usually large); and $g(\chi ) = \lambda_{1} \cdot TGV_{\alpha }^{2} (\chi )$ or $\lambda_{2} \varphi (\chi )$ is a continuous convex function which is nonsmooth. According to the FISTA algorithm, given a continuous function *g*(*u*) and any scalar *L* > 0, the proximal map associated with function $g(\chi )$ can be built as follows:

5 $$prox_{L} \{ g(u),\chi \} = \arg \mathop {\min }\limits_{u} \{ g(u) + \frac{L}{2}\left\| {u - \chi } \right\|^{2} \}$$

Equations (2) and (3) are solved in an iterative fashion. Let ***X***_**1**_ be the solution of the TGV subproblem Eq. (2) and ***X***_**2**_ be the solution of the TD subproblem Eq. (3) respectively; in each *k* iteration, the solutions $\chi_{k}$ to the overall problem Eq. (1) can be found by a linear combination as follows:

6 $$\chi_{k} = \frac{1}{2}(X_{1} + X_{2} )$$

The FCSA-based algorithm for solving the *k-t* TGV-TD problem-based CS-*d*MRI reconstruction can be described in the algorithm 1.
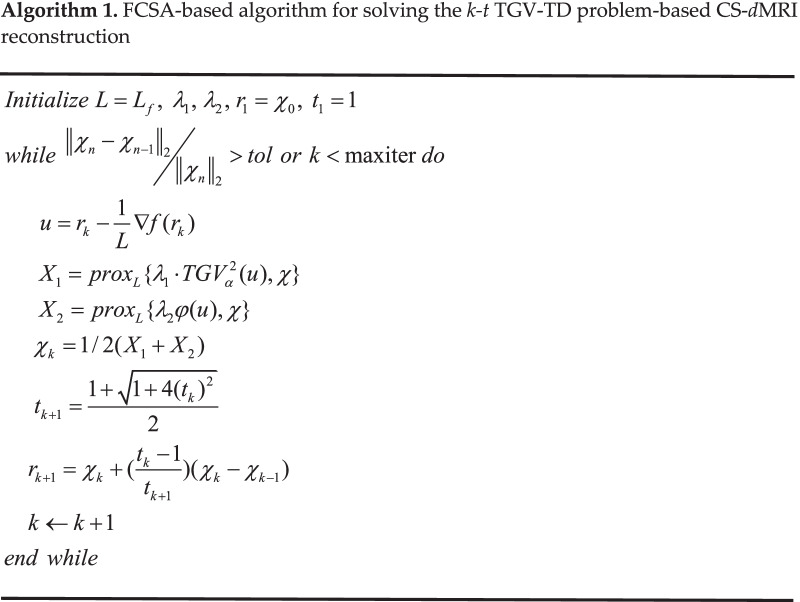


## Materials and methods

To test the reconstruction performance of the proposed *k-t* TGV-TD method, two different MR raw datasets, *i.e.* cardiac perfusion and cardiac cine, were employed in this study. The cardiac perfusion MR datasets was obtained on a 3 T Siemens scanner with saturation recovery sequence (TR/TE = 2.5/1 ms, saturation recovery time = 100 ms) at the University of Utah [30]. It contained 70 frames, and the data from a single slice was acquired on a Cartesian grid with a k-space matrix of 90 × 190 (phase encoding × frequency encoding) at a temporal resolution of one heartbeat. The cardiac cine MR data was acquired on a 1.5 T Philips system at Yonsei University Medical Center [41, 42]. The dataset is composed of 25 frames of full k-space data. The matrix size for scanning is 256 × 256, which corresponds to 256 phase encoding steps and 256 samples in frequency encoding. The cardiac cine dataset was obtained using steady-state free precession (SSFP) sequence with a flip angle of 50 degree and TR = 3.45 ms. The field of view was 345 mm × 270 mm, and the slice thickness was 10 mm. In addition, the radial sampling pattern was used to undersample the k-space of these two datasets, which was simulated by rounding the sample locations to the nearest Cartesian location [30, 41].

In each experiment, the regularization parameters λ_1_ and λ_2_ in the problem (1) were determined by parameter sweeping. The following stopping criteria were adopted for all experimental settings: the tolerance as shown in the FCSA-based reconstruction algorithm was set as *tol* = 10^–4^, and the maximum number of iterations was 30. All reconstructions were implemented in the Matlab programming environment (Version 2017b, Mathworks, Natick, MA), and the experiments were performed on a personal computer with 3.6 GHz Intel Core i9-9900 K CPU, 32 GB of memory and Windows 10 operating system. In addition, we compared the proposed *k-t* TGV-TD reconstruction method with four state-of-the-art dynamic CS-MRI reconstruction methods, that is, HOSVD-based tensor decomposition method [37, 43], *k-t* SLR [30], 3D-TGV, and low-rank plus sparse method [44].

To further quantitatively evaluate the reconstruction methods, the peak SNR (PSNR) and structural similarity index (SSIM) were adopted in this work [45]. Furthermore, the reconstructed images and the corresponding error images (the absolute difference between reconstructed image and the full sampled MR image) were also compared visually. The PSNR and SSIM were formulated as follows:

7 $$PSNR = 20\log_{10} \left( {{{MAX_{{I_{ref(r)} }} } \mathord{\left/ {\vphantom {{MAX_{{I_{ref(r)} }} } {\sqrt {\frac{1}{mn}\sum\nolimits_{i = 1}^{m} {\sum\nolimits_{j = 1}^{n} {\left( {I_{ref} (r) - I(r)} \right)} } } }}} \right. \kern-\nulldelimiterspace} {\sqrt {\frac{1}{mn}\sum\nolimits_{i = 1}^{m} {\sum\nolimits_{j = 1}^{n} {\left( {I_{ref} (r) - I(r)} \right)} } } }}} \right)$$

8 $$SSIM\left( {I_{{{\text{ref}}}} ,I} \right) = {{\left( {2\mu_{{I_{ref} }} \mu_{I} + c_{1} } \right)\left( {2\sigma_{{I_{ref} I}} + c_{2} } \right)} \mathord{\left/ {\vphantom {{\left( {2\mu_{{I_{ref} }} \mu_{I} + c_{1} } \right)\left( {2\sigma_{{I_{ref} I}} + c_{2} } \right)} {\left( {\left( {\mu_{{_{{I_{ref} }} }}^{2} + \mu_{{_{I} }}^{{^{2} }} + c_{1} } \right)\left( {\sigma_{{I_{ref} }}^{2} + \sigma_{I}^{2} + c_{2} } \right)} \right)}}} \right. \kern-\nulldelimiterspace} {\left( {\left( {\mu_{{_{{I_{ref} }} }}^{2} + \mu_{{_{I} }}^{{^{2} }} + c_{1} } \right)\left( {\sigma_{{I_{ref} }}^{2} + \sigma_{I}^{2} + c_{2} } \right)} \right)}}$$

where $MAX_{{I_{ref(r)} }}$ is the maximum signal intensity of $I_{ref} (r)$. $\mu_{{I_{ref} }}$ and $\mu_{I}$ are mean signal values of $I_{ref} (r)$ and $I(r)$, $\sigma_{{I_{ref} }}^{2}$ and $\sigma_{I}^{2}$ are variance of $I_{ref} (r)$ and $I(r)$, $\sigma_{{I_{ref} I}}$ is the covariance of $I_{ref} (r)$ and $I(r)$, $c_{1}$ and $c_{2}$ are two variables to stabilize the equation when the denominator is too small.

## Results

### Comparisons on the cardiac perfusion dataset

**Fig. 1:**
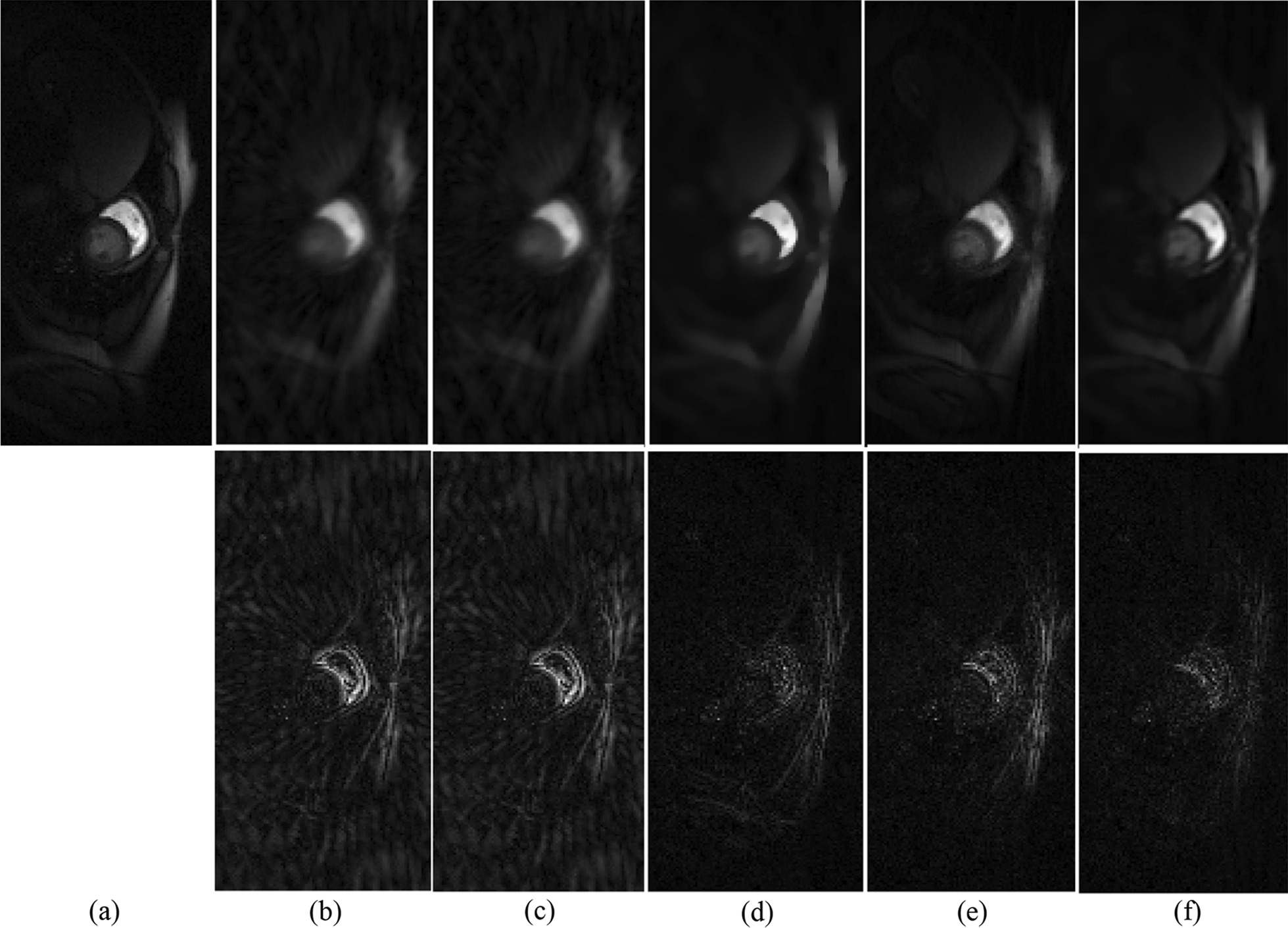
Performance evaluation of *k-t* TGV-TD method in comparison with HOSVD, *k-t* SLR, 3D-TGV, and L + S methods on the cardiac perfusion dataset. The first row is the reconstructed MR image with acceleration factor 6; the second row is the error map. **a** MRI reconstructed with fully sampled k space data; **b**–**f** reconstructed MRI and the corresponding error images by using HOSVD, *k-t* SLR, 3D-TGV, L + S, and *k-t* TGV-TD respectively

The proposed *k*-*t* TGV-TD method was employed to reconstruct the cardiac perfusion data with variously reduced k-space sampling data. Figure 1 displays the performance evaluation of *k*-*t* TGV-TD method in comparison with HOSVD, *k-t* SLR, 3D-TGV, and L + S [44] methods on the cardiac perfusion MR dataset with acceleration factor 6. The reconstructed MR image of one representative frame (13th frame) and the error map were provided as a comparison. As can be seen in Fig. 1, the proposed *k-t* TGV-TD method outperformed the HOSVD, k-t SLR, 3D-TGV and L + S methods in reducing artifacts, which was presented clearly in the error maps (the second row).

**Fig. 2:**
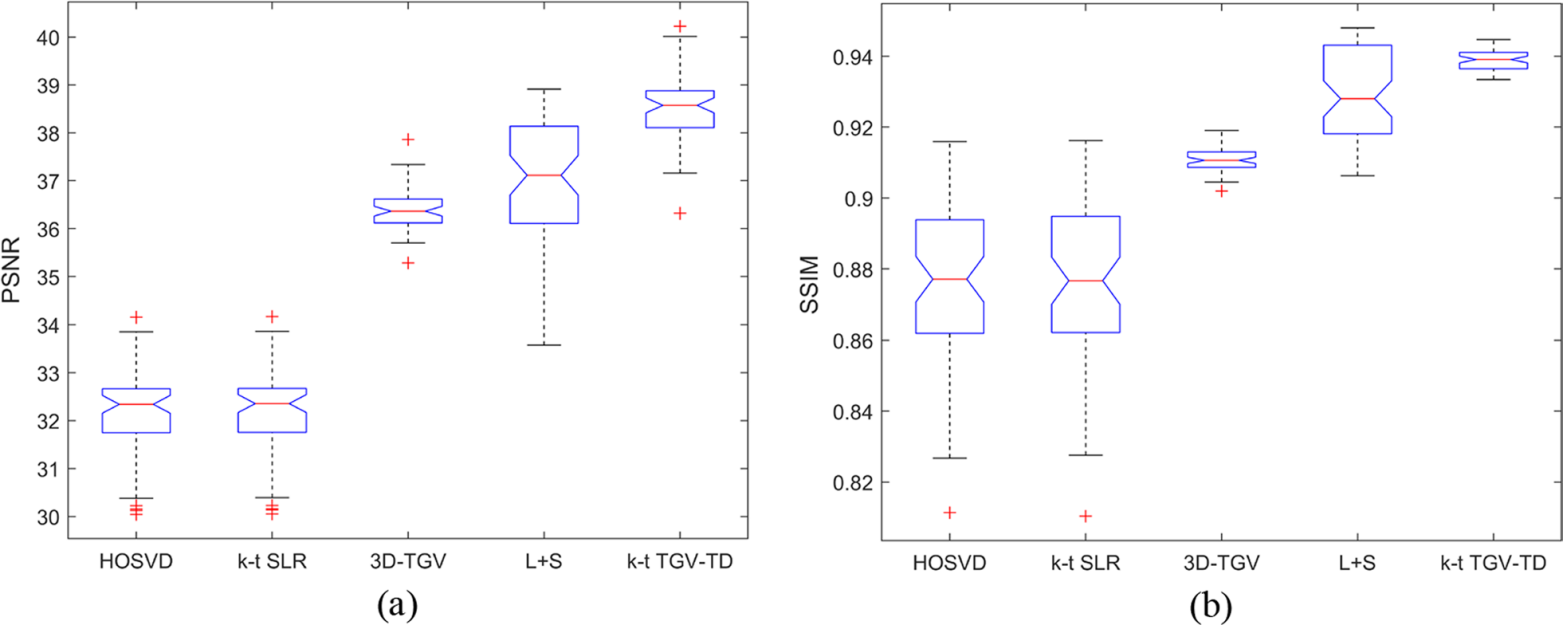
The PSNR and SSIM of the reconstructed cardiac perfusion images by using HOSVD, *k-t* SLR, 3D-TGV, L + S, and *k-t* TGV-TD, with acceleration factor 6. **a** PSNR; **b** SSIM

As listed in Table 1, the PSNR and SSIM of cardiac perfusion dataset were provided by using HOSVD, *k-t* SLR, 3D-TGV, L + S, and *k-t* TGV-TD with acceleration factors: 6, 8, and 10 respectively. It can be found that, compared with the other methods, the proposed *k-t* TGV-TD method can achieve significantly better reconstruction results in terms of PSNR and SSIM. In addition, notched box plots about PSNR and SSIMR comparison among the above five methods were provided for cardiac perfusion datasets, as shown in Fig. 2. Since the notches in the box plot do not overlap, it is concluded that, the proposed *k-t* TGV-TD method outperforms state-of-the-art methods with 95% confidence. Meanwhile, PSNR and SSIM of the reconstructed MR images by using *k-t* TGV-TD method were more stable than those by HOSVD, *k-t* SLR, and L + S methods.

**Table 1:** Comparisons in terms of PSNR and SSIM of different acceleration factors on the cardiac perfusion dataset

Methods	Acceleration factor					
	6		8		10	
	PSNR	SSIM	PSNR	SSIM	PSNR	SSIM
HOSVD	32.07	0.8700	30.75	0.8017	29.36	0.7301
*k-t* SLR	32.08	0.8705	30.81	0.8056	29.64	0.7465
3D-TGV	36.42	0.9108	35.72	0.9021	34.78	0.8906
L + S	36.94	0.9306	36.16	0.9221	35.42	0.9136
*k-t* TGV-TD	38.66	0.9392	37.08	0.9315	36.10	0.9210

### Comparisons on the cardiac cine dataset

**Fig. 3:**
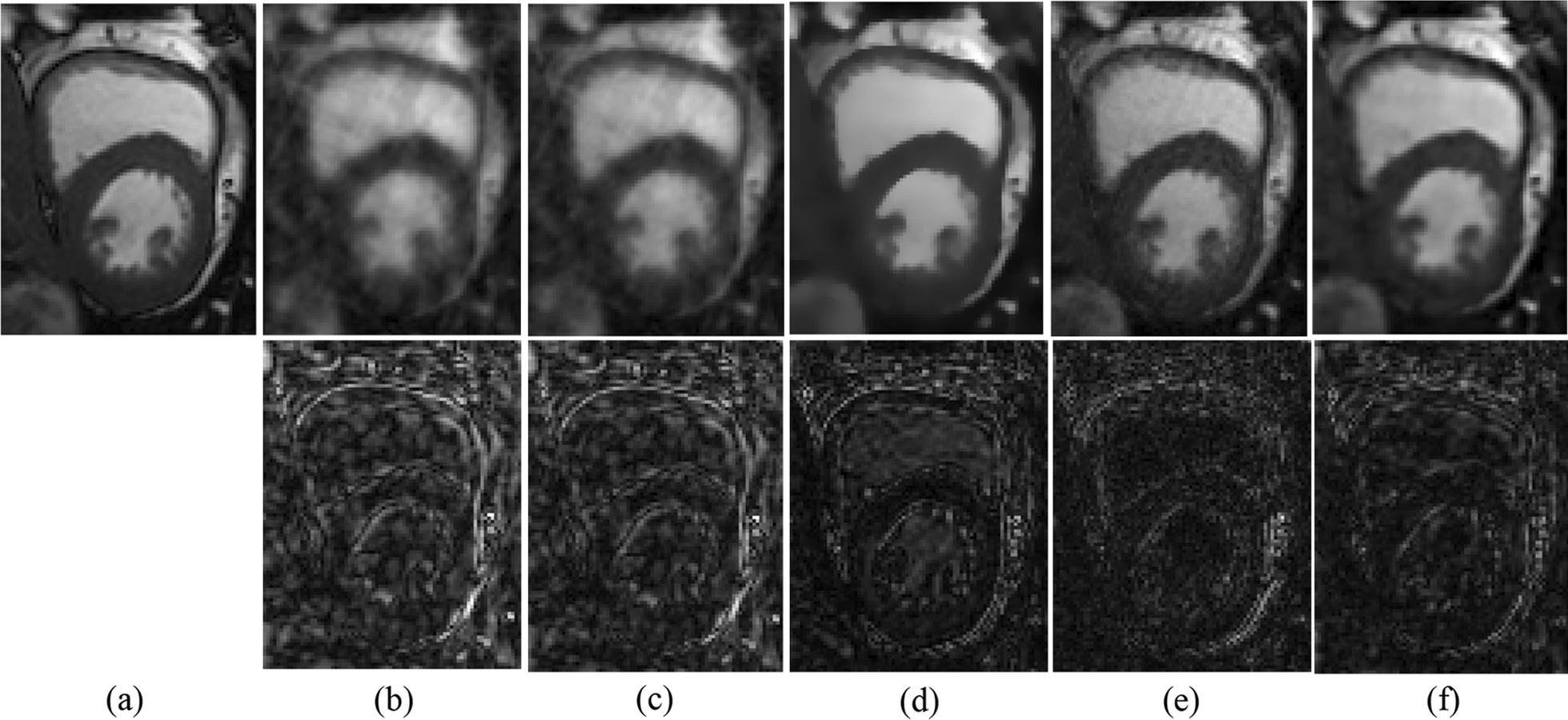
Performance evaluation of *k-t* TGV-TD method in comparison with HOSVD, *k-t* SLR, 3D-TGV, and L + S methods on the cardiac cine dataset. The first row is the reconstructed MR image with acceleration factor 6; the second row is the error map. **a** MRI reconstructed with fully sampled k-space data; **b**–**f** reconstructed MRI and the corresponding error images by using HOSVD, *k-t* SLR, 3D-TGV, L + S, and *k-t* TGV-TD respectively

Figure 3 shows the visual comparisons of the reconstructed results by the proposed *k-t* TGV-TD method, HOSVD, *k-t* SLR, 3D-TGV, and L + S methods with radial sampling pattern at reduction factor 6. The first row was the reconstructed MRI (13th frame of the cardiac cine data), and the second row showed the error map. Visually, as shown in Fig. 3, one can find that the proposed method outperformed the HOSVD, *k-t* SLR, 3D-TGV, and L + S methods in reconstructing MRI with better defined borders and less reconstruction artifacts. Reconstructions by using the HOSVD and *k-t* SLR methods were contaminated by aliasing artifacts and noises, and the image reconstructed by using 3D-TGV method was over-smoothed.

**Fig. 4:**
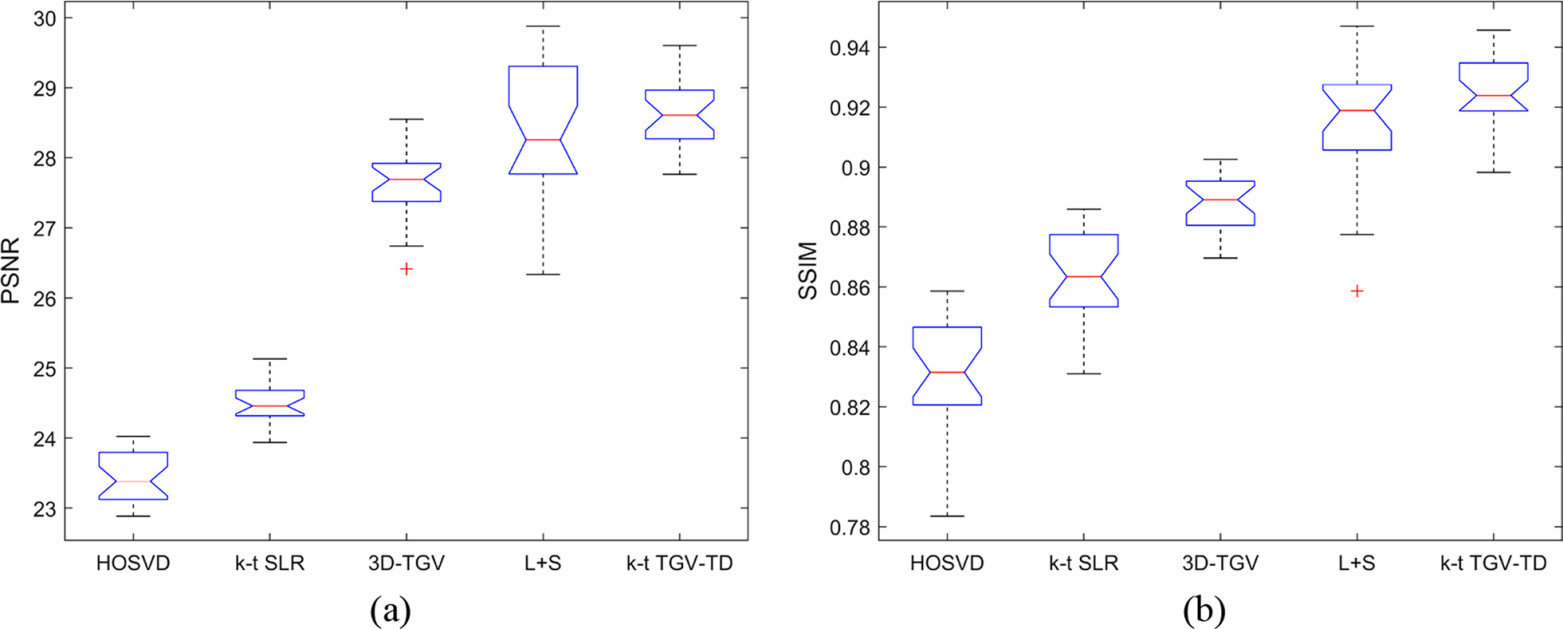
The PSNR and SSIM of the reconstructed cardiac cine images by using HOSVD, *k-t* SLR, 3D-TGV, L + S, and *k-t* TGV-TD, with acceleration factor 6. **a** PSNR; **b** SSIM

As listed in Table 2, the PSNR and SSIM of cardiac cine dataset were provided by using HOSVD, *k-t* SLR, 3D-TGV, L + S, and *k-t* TGV-TD with acceleration factor: 4, 6, and 8 respectively. It can be found that, compared with the other methods, the proposed *k-t* TGV-TD method can achieve better reconstruction results in terms of PSNR and SSIM. In addition, notched box plots about PSNR and SSIMR comparison among the above five methods with acceleration factor 6 were provided for cardiac cine datasets, as shown in Fig. 4. It can be found obviously that the proposed *k-t* TGV-TD method outperforms the other methods with higher PSNR and SSIM in most of the frames.

**Table 2:** Comparisons in terms of PSNR and SSIM of different acceleration factors on the cardiac perfusion dataset

Methods	Acceleration factor					
	4		6		8	
	PSNR	SSIM	PSNR	SSIM	PSNR	SSIM
HOSVD	26.25	0.9182	23.43	0.8298	21.59	0.7569
*k-t* SLR	27.90	0.9412	24.47	0.8632	22.54	0.7948
3D-TGV	28.92	0.9227	27.63	0.8880	24.53	0.8209
L + S	31.07	0.9520	28.19	0.9137	26.87	0.8857
*k-t* TGV-TD	31.13	0.9586	28.60	0.9249	26.88	0.8994

## Discussion

In this work, based on combination of the tensor decomposition and 3D-TGV method, the *k-t* TGV-TD method was proposed to reconstruct highly undersampled cardiac perfusion and cine MR images. The tensor decomposition-based sparsity regularization method exploited both the intra and inter sparsity of each frame, therefore it was an effective way to make use of the three-dimensional redundancy in dynamic cardiac datasets. Moreover, the TGV method can effectively alleviate the staircase artifacts of TV based MR image reconstruction, and the 3D-TGV method can further apply the sparsity between and within the frames to improve the reconstruction accuracy.

From the reconstruction results, as shown in Figs. 1 and 3, it can be observed that the proposed method can outperform the HOSVD, *k-t* SLR, 3D-TGV and L + S methods in the investigated different dynamic cardiac datasets. The *k-t* TGV-TD method can reconstruct the MR images with less error artifacts than those by using HOSVD, *k-t* SLR, 3D-TGV and L + S methods. From the quantitative evaluation indexes PSNR and SSIM, advantages of the proposed *k-t* TGV-TD method over other methods were apparent.

In this work, the proposed *k-t* TGV-TD method explored the correlations and sparsity of the dynamic cardiac datasets, but did not integrate with partially parallel imaging (PPI). In addition, a combination of compressed sensing and parallel imaging was proposed to reconstruct the MR image [46, 47], which can further reduce the k-space acquisition. In the future work, we will consider combining the *k-t* TGV-TD method with the PPI reconstruction method to further improve dynamic cardiac MR image reconstruction quality at higher reduction factors.

## Conclusion

In this paper, a novel technique, termed *k-t* TGV-TD, that combines the tensor decomposition and 3D total generalized variation, was proposed for dynamic cardiac MR imaging reconstruction. The method was evaluated with cardiac perfusion and cine datasets. The experimental results indicated that, compared with the HOSVD, *k-t* SLR, 3D-TGV and L + S methods, the proposed *k-t* TGV-TD method could achieve improved reconstruction accuracy in all the cases under investigation.

## Data Availability

The cardiac perfusion data was acquired at the University of Utah and publicly available via web-link: http://www.engineering.uiowa.edu/~jcb/Software/ktslr_matlab/Software.html. The MR data were acquired at Yonsei University Medical center in Korea and are publicly available via the web-link: https://github.com/jasonbme/k-t-focuss

## Abbreviations

MRI: Magnetic Resonance Imaging
DCMRI: Dynamic cardiac MR imaging
CS: Compressed sensing
dMRI: Dynamic magnetic resonance imaging
HOSVD: High order singular value decomposition
TV: Total variation
TGV: Total generalized variation
DCT: Discrete cosine transform
DWT: Discrete wavelet transform
SVD: Singular value decomposition
HDTV: Higher degree total variation
NLTV: Nonlocal total variation
*k-t* SLR: K-t sparisity and low-rank method
FCSA: Fast composite splitting algorithm
SSFP: Steady-state free precession
FOV: Field of view
FISTA: Fast iterative shrinkage-threshold algorithm
